# Risk profiles of lipids, blood pressure, and anthropometric measures in childhood and adolescence: project heartBeat!

**DOI:** 10.1186/s40608-016-0090-8

**Published:** 2016-02-18

**Authors:** Edward Haksing Ip, Xiaoyan Leng, Qiang Zhang, Robert Schwartz, Shyh-Huei Chen, Shifan Dai, Darwin Labarthe

**Affiliations:** Department of Biostatistical Sciences, Wake Forest School of Medicine, Winston-Salem, NC USA; Department of Pediatrics, Wake Forest School of Medicine, Winston-Salem, NC USA; Division of Nutrition, Physical Activity and Obesity, Center for Disease Control and Prevention, Atlanta, GA USA; Department of Preventive Medicine, Feinberg School of Medicine, Northwestern University, Evanston, IL 60208 USA

**Keywords:** Cardiovascular risk profiles, Health factors, Multivariable health factor profiles, Abdominal obesity, Principal component functional curve, NHANES III

## Abstract

**Background:**

Many common risk factors for cardiovascular disease (CVD) originate in childhood and adolescence. There is a lack of literature examining variability within study populations, as well as a shortage of simultaneous analyses of CVD risk factors operating in tandem.

**Methods:**

We used data from Project HeartBeat!-a multi-cohort longitudinal growth study of children and adolescents in the US - for assessing multiple profiles for lipids, blood pressure, and anthropometric measures. Principal component functional curve analysis methods were used to summarize trajectories of multiple measurements. Subsequently less favorable health (high risk) and more favorable (low risk) groups from both female and male cohorts were identified and compared to US national norms.

**Results:**

Compared to national norms, the high risk groups have increased waist circumference, body mass index, and percent body fat as well as higher low-density lipoprotein cholesterol and triglyceride levels, and lower high-density lipoprotein cholesterol. The risk profiles also exhibit patterns of convergence and divergence across the high and low risk groups as a function of age.

**Conclusions:**

These observations may have clinical and public health implications in identifying groups of children at high risk of CVD for earlier interventions.

## Background

Cardiovascular diseases (CVDs) often do not become clinically manifest until adulthood. The cumulative effects of risk factors including abdominal obesity, elevated body mass index (BMI) levels, dyslipidemia, and hypertension originating in childhood may become clinically manifested as coronary heart disease, heart failure, and stroke [1–8]. Results from the Muscatine Study suggest that previous values of lipid or blood pressure levels for individuals at younger ages were strong predictors of the values for individuals aged 20–39 years. Abdominal obesity in particular often precedes other components of the metabolic syndrome, and may play a role in the development of other cardiovascular risk factors such as hypertension [7, 9]. The cumulative life-course burden of excessive body fat, as indicated by measures including waist circumference, BMI, and percent body fat, begins at an early age. These effects may transpire into cardiovascular events in adulthood, either through major risk factors or, independent of them [9, 10].

While previous reports have described a broad picture of the growth process on lipids, blood pressure, and anthropometry in adolescents and young adults across gender and race subgroups [11–24], there are significant questions that have not been directly addressed. First, substantial variability exists in almost all of the lipid, blood pressure, and anthropometric measures. Even when the data are examined cross-sectionally, population differences in cholesterol concentrations at a given age were as great as 40–50 mg/dL [23]. Additionally, temporal patterns differ significantly from individual to individual within the same age-race subgroup.

**Fig. 1:**
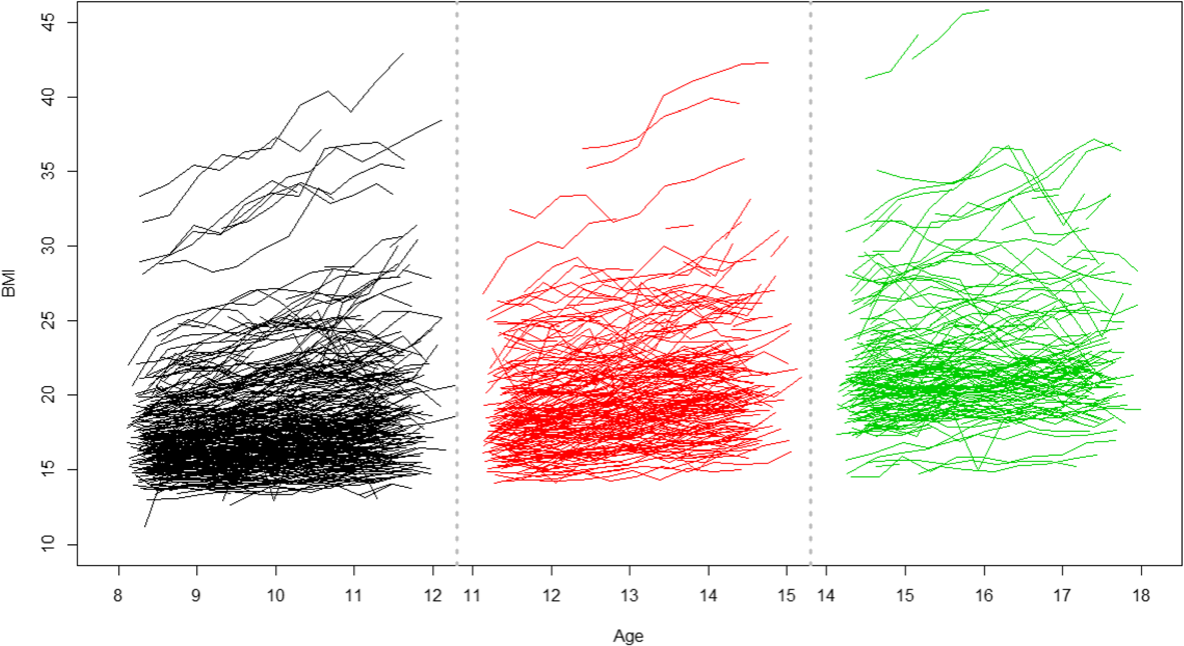
Trajectories of the three cohorts for females in BMI (kg/m^2^), Project HeartBeat!

Typically, temporal patterns were depicted as smoothed mean curves that were based on quadratic and cubic polynomials for describing the change of lipids, blood pressure, and anthropometry over the target age range [16]. Fig. 1 shows the growth trajectories of BMI using Project HeartBeat! data, which were also used in the current analysis. The often-substantial variations across individuals in the temporal patterns are not captured by a population-averaged, model-based growth curve. It is possible that subgroups of different risk strata exist within the population. From a prevention point of view, these subgroups need to be identified and carefully characterized.

A second question that has not been adequately addressed in the prior literature concerning cardiovascular risk in childhood and adolescence is the lack of analysis of multiple profiles across lipids, blood pressure, and anthropometry. Previous analysis has focused on single-variable trajectories and has thus missed the opportunity to examine multiple trajectories of cardiovascular outcomes from the same group of individuals across an age span. For example, if the cholesterol trajectory of a high-risk group suggests that cholesterol tends to increase at an accelerated pace, then it would be natural to ask whether or not the blood pressure of the same group of individuals also increases over time.

The purpose of this study was to (1) analyze multiple profiles of lipids, blood pressure, and anthropometry using Project HeartBeat! data, and (2) identify subgroups with different cardiovascular risks. By understanding the overall risk patterns that appear during early life, the findings in this study could lead to early-life interventions for reducing or delaying adverse metabolic changes and cardiovascular outcomes.

## Methods

### Data and measures

Project HeartBeat! is a population-based epidemiologic study that examines early development of cardiovascular risk factors in anthropometry, blood pressure, and lipids as a growth process. Using intensive, longitudinal follow-up assessments of children and adolescents, the study collected data that offer a rich resource for determining the cardiovascular profiles of individuals aged 8–18 and their concurrent association with body size and composition. The study contained three separate cohorts that were monitored for 4 years: cohort 1 of 8–12 years old; cohort 2 of 11–15 years old; and cohort 3 of 14–18 years old, with a total sample size of *N* = 678 (49 % female, 20 % Black, total number of observations = 5500).

Data collection took place from October 1991 through August 1995. Each participant was scheduled for examination three times each year. In the original study, the University of Texas Health Science Center at Houston and the Baylor College IRBs approved data collection and the proposed research. For the purposes of this analysis, only data collected during the annual anniversary examinations, of which the percentages of missing values were minimal, were used. To facilitate comparison to previous results, eight health measures used in previous studies were used in this study. The measures include waist circumference (WC, in cm), BMI, percent body fat (PBF, in %), low-density lipoprotein cholesterol (LDL-C, in mg/dl), high-density lipoprotein cholesterol (HDL-C, in mg/dl), triglyceride (in mg/dl), and phase 4 diastolic and systolic blood pressure (DBP and SBP, in mmHg). Other potential cardiovascular risk variables such as energy intake and physical activity have not been included for comparability as well as technical reasons. Inclusion of more variables, for example, tends to lead to an increased number of only slightly different cardiovascular profiles, making meanginful interpretation of results challenging, Details of the study rationale, design, and data collection procedures have been described elsewhere [24].

### Statistical methods

Figure 1 shows the raw data trajectories for females for one variable – BMI – in the three cohorts. For each cohort, we treated the repeated measurements (e.g., BMI) of an individual as constituting a functional curve [25–27]. Therefore the multivariate approach implied that each individual within an age cohort had a total of 8 functional curves to be simultaneously analyzed.

The analytic method comprised three steps: (1) functional principal component (FPC) analysis for summarizing the information contained in the functional curves into a few numbers, called FPC scores [26, 27]; (2) mixture modeling, or latent class analysis [28], for the FPC values derived from the different variables to delineate relatively homogeneous subgroups of individuals in terms of their FPC values; and (3) projection of the FPCs onto the original variable spaces to show the group-based functional curves in their original scales because FPC were combinations of the original variables and were difficult to interpret. We used the Akaike Information Criterion (AIC), a commonly used goodness-of-fit index, in selecting the number of clusters (latent classes) in step (2). The statistical testing of the difference between group-based functional curves, or trajectories, was based on 95 % confidence bands of the mean group trajectories.

In order to compare the group-based functional curves with national norms, we also included gender-specific population-level growth trajectories into our analysis using NHANES III data. The NHANES III is a nationally representative cross-sectional survey conducted during the period of 1988 to 1994 [29, 30], which is contemporaneous with the HeartBeat! data collection phase. Except for PBF, the NHANES III mean values reported in this work were derived from raw data. In order to account for the complex sampling design, proper sampling weights for measurements collected from Mobile Examination Centers (MECs) were used in our derivation. The mean values of PBF for some age groups were adapted from a published reference [31]. Related national statistic for the measures used in this study and related health guidelines can be found in other references [32–35]. All analyses were performed in 2014–2015.

## Results

**Fig. 2:**
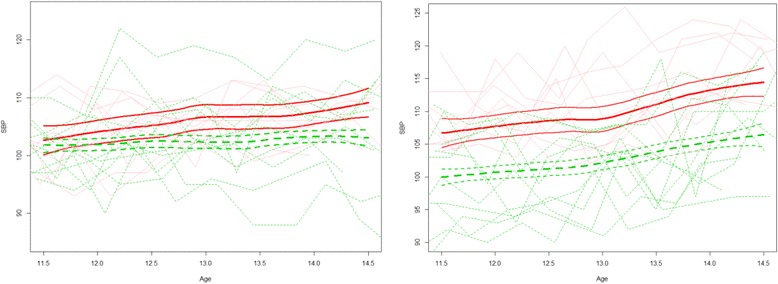
Examples of mean trajectories and 95 % confidence bands of the less favorable cardiovascular health group (LF-CHG, *solid lines*) and more favorable cardiovascular health group (MF-CHG, *dashed lines*) for statistically non-significant different groups (left panel, SBP of 11-year old female cohort) and statistically significant groups (right panel, SBP of 11-year old male cohort). For each group, a random sample (*n* = 10) of individual trajectories are shown in *shaded color* in background

**Fig. 3:**
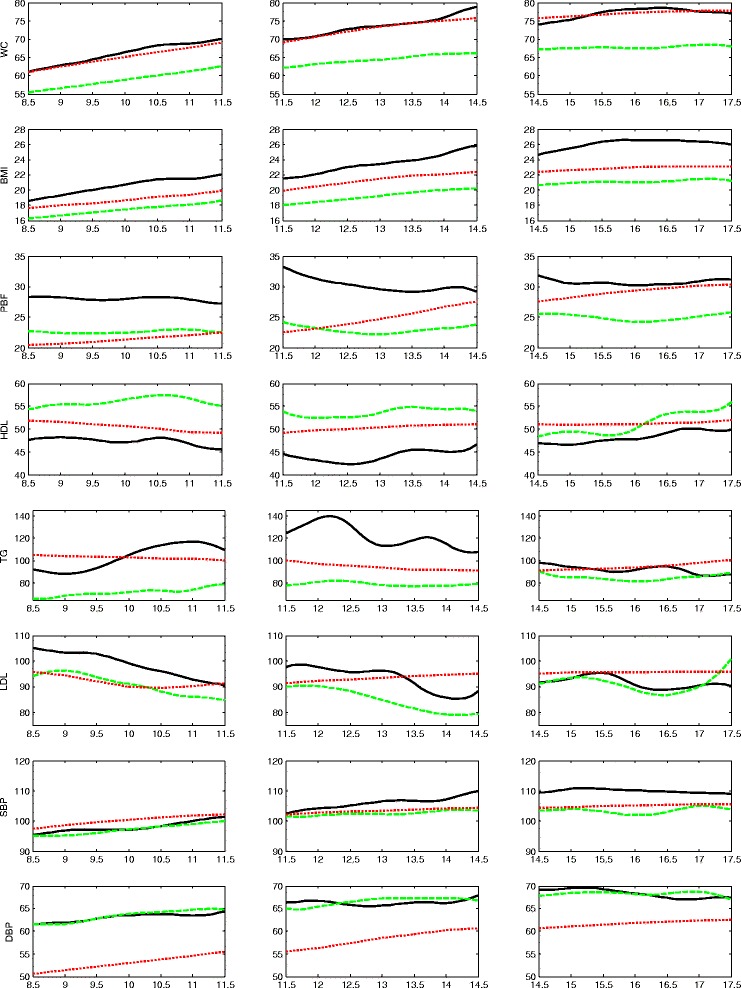
Trajectories of the less favorable cardiovascular health group (LF-CHG, *solid lines*) and more favorable cardiovascular health group (MF-CHG, *dashed lines*) for the female cohorts. The national mean values derived from NHANES III are shown as *dotted lines*. The NHANES III curves have been smoothed. Three age cohorts are shown separately in three column panels

**Fig. 4:**
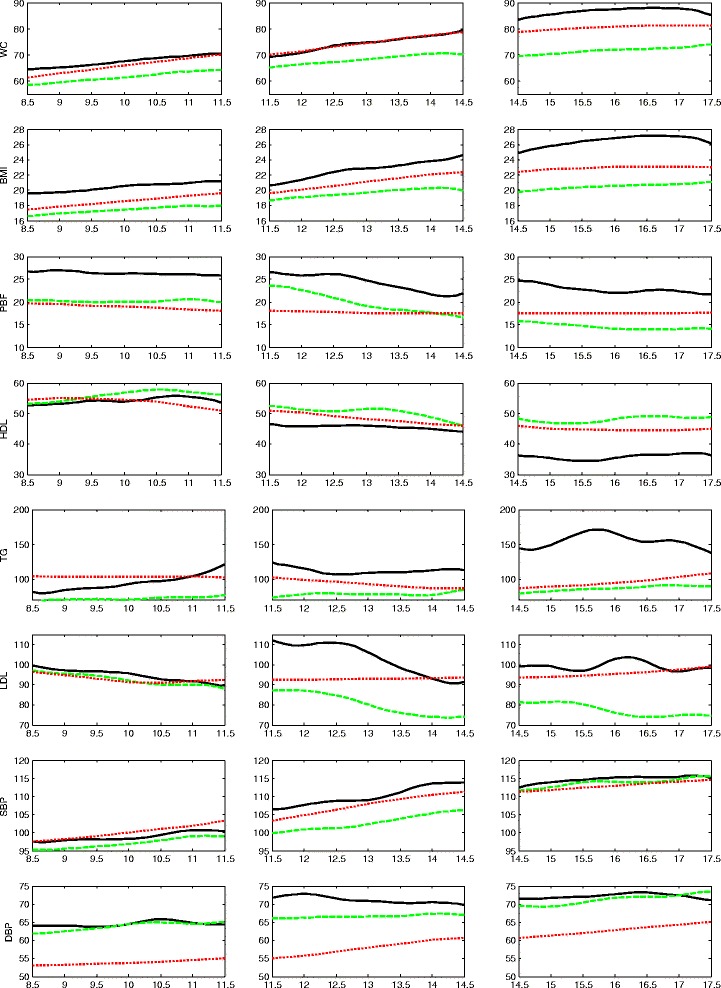
Trajectories of the less favorable cardiovascular health group (LF-CHG, *solid lines*) and more favorable cardiovascular health group (MF-CHG, *dashed lines*) for the male cohorts. The national mean values derived from NHANES III are shown as *dotted lines*. The NHANES III curves have been smoothed. Three age cohorts are shown separately in three column pane

Based on the AIC, 2-cluster solutions were selected for each age-gender cohort. The AIC values were close for 1-cluster and 2-cluster solutions for females in cohort two, but the 2-cluster solution was ultimately favored. Based on the overall lipid, blood pressure, and anthropometry outcomes, we labeled the two clusters less favorable cardiovascular health group (LF-CHG) and more favorable cardiovascular health group (MF-CHG). Note that the MF- and LF-CHG were not defined by pre-specified cut points; group membership was determined through latent class analysis and the labels were assigned based on inspection of the respective group trajectories. Figure 2 shows two examples of the mean trajectories of the MF- and LF-CHG and their respective 95 % confidence bands. When two confidence bands on a specific variable were completely separate and did not overlap, statistical significance between the MF- and LF-CHG was declared for the variable (*p* < .05). On the other and, if any part of the group trajectories overlapped, then the groups were deemed not statistically significant (*p* > .05). Table 1 shows the descriptive statistics for the health groups. Figures 3 (females) and 4 (males) show the averaged functional curves of the LF-CHG and the MF-CHG across the 8 designated measures. The functional curves of the three cohorts are separately depicted. To facilitate reading, the LF-CHG is indicated by a solid line and the MF-CHG is indicated by a dashed line in each cohort. The values in anthropometry, lipid level, and blood pressure functional curves for the LF-CHG and MF-CHG were contrasted and also compared with NHANES III data for children aged 8–18 years old. Smoothed curves for national data means were depicted as dotted lines in Figs. 3 and 4. Specifically, the local polynomial regression fitting (LOESS) procedure [36], as implemented in the function loess() in R with smoothing parameters set at default values, was used for smoothing. Table 2 summarizes the statistical testing results between the mean trajectories of MF-CHG and the LF-CHG. As Figs. 3 and 4 visualize the results and Table 1 summarizes testing, in the Result section we only highlight the most salient findings.

**Table 1:** Sample size and participant characteristics: Project HeartBeat!

	8 year old cohort		11 year old cohort		14 year old cohort	
	MF-CHG^a^	LF-CHG^a^	MF-CHG	LF-CHG	MF-CHG	LF-CHG
Males						
n (%)	110 (72.8 %)	41 (27.2 %)	70 (72.2 %)	27 (27.8 %)	55 (77.5 %)	16 (22.5 %)
Race (n,%)						
White	81 (75.8 %)	26 (24.3 %)	53 (72.6 %)	20 (27.4 %)	44 (73.3 %)	16 (26.7 %)
Black	22 (61.1 %)	14 (38.9 %)	12 (63.2 %)	7 (36.8 %)	4 (100 %)	0 (0 %)
Non-Black	7 (87.5 %)	1 (12.5 %)	5 (100 %)	0 (0 %)	7 (100 %)	0 (0 %)
Age (years)^b^	8.46	8.62	11.5	11.51	14.38	14.45
Females						
n (%)	90 (62.1 %)	55 (37.9 %)	69 (81.2 %)	16 (18.8 %)	50 (64.9 %)	27 (35.1 %)
Race (n,%)						
White	64 (62.1 %)	39 (37.9 %)	55 (82.1 %)	12 (17.9 %)	45 (71.4 %)	18 (28.6 %)
Black	22 (59.5 %)	15 (40.5 %)	11 (78.6 %)	3 (21.4 %)	4 (40.0 %)	6 (60.0 %)
Non-Black	4 (80.0 %)	1 (20.0 %)	3 (75.0 %)	1 (25 %)	1 (25 %)	3 (75.0 %)
Age (years)	8.54	8.51	11.5	11.54	14.44	14.48

^a^ *MF-CHG* More favorable cardiovascular health group. *LF-CHG* Less favorable cardiovascular health group

^b^mean value

**Table 2:** Result for statistical tests between LF-CHG and MF-CHG mean trajectories at the significance level of 0.05

	WC	BMI	PBF	HDL	Triglyceride	LDL	SBP	DBP
Females								
8 year old cohort	<.05	<.05	<.05	<.05	<.05			
11 year old cohort	<.05	<.05	<.05	<.05	<.05			
14 year old cohort		<.05	<.05					
Males								
8 year old cohort	<.05	<.05	<.05					
11 year old cohort	<.05	<.05	<.05		<.05	<.05	<.05	
14 year old cohort	<.05	<.05	<.05	<.05	<.05	<.05		

### Results for the three female cohorts

For the first cohort ( age 8–12 years) , 38 % (55/145) of females belong to the LF-CHG, which is characterized by an overall higher WC, higher BMI, higher PBF, higher level of triglyceride, lower HDL-C, and higher LDL-C (solid lines in Fig. 3 leftmost panels), as compared to the MF-CHG (dashed lines). Waist circumference (WC) in the MF-CHG female cohort one (~55 cm at age 8 and ~63 cm at age 12) is lower than the mean value obtained from NHANES III (60.9 cm at age 8 and 72.9 cm at age 12) whereas WC in the LF-CHG is comparable (~62 cm at age 8 and 70 cm at age 12). For BMI, the NHANES III mean values for females 8–12 range from 17.7 kg/m^2^ (age 8) to 21.5 kg/m^2^ (age 12). The LF-CHG had a higher BMI (~19 kg/m^2^ at age 8 and 22 kg/m^2^ at age 12). The separation of PBF between LF-CHG (~28 %) and MF-CHG (~23 %) is also pronounced in this age cohort. Trajectories of LF-CHG and MF-CHG for HDL-C, LDL-C, and triglycerides are separated, with the LDL-C levels showing slightly declining trends.

Blood pressure levels in both the LF-CHG and MF-CHG generally trend with the NHANES III averages but the DBP levels are ~10 mmHg above. However, the trend lines for both DBP and SBP are not clearly separated between the LF-CHG and MF-CHG.

For the second cohort (age 11–15 years), 19 % (16/85) of females are identified as LF-CHG (solid lines in Fig. 3, middle panels). Perhaps with the exception of DBP, the two groups appear to be distinct across the various measures. Compared with the MF-CHG, the LF-CHG has higher WC, BMI, PBF; higher triglyceride, higher LDL-C, lower HDL-C, and higher SBP. BMI values of the MF-CHG is lower than the NHANES III average, which starts at 17.5 kg/m^2^ (age 11) and ends at 20 kg/m^2^ (age 15), while the BMI of the LF-CHG is ~2 to 3 kg/m^2^ above the NHANES III average. The HDL-C and LDL-C levels of both the LF-CHG and MF-CHG are within an acceptable range but the LF-CHG has an elevated level of triglyceride (>110 mg/dl) almost throughout the entire age range, suggesting that the LF-CHG is either at the borderline-high (90–129 mg/dl) or high level (130 mg/dl). The SBP between the two groups tends to slightly diverge, with the LF-CHG trending higher, but DBP levels are not well separated.

For the third cohort (age 14–18 years), the LF-CHG constitutes approximately 35 % (27/77) of the cohort (solid line in Fig. 3, rightmost panels). The most striking feature is that the anthropometric measures—WC, BMI, and PBF—all tend to be well separated for the LF-CHG and MF-CHG while lipid measures—LDL-C, HDL-C, and triglyceride—all tend to converge between the two groups.

The levels of lipids in this cohort are not very well separated between the LF-CHG and MF-CHG. For SBP, the MF-CHG is approximately 5 mmHg lower than the LF-CHG, of which the values are comparable to the NHANES III mean values of this age range (105–110 mmHg).

### Results for the three male cohorts

For the first cohort of males, 27 % (41/151) of the males are identified as belonging to the LF-CHG, which exhibits higher BMI, WC, and PBF; higher triglyceride (solid lines in Fig. 4, leftmost panels). The PBF of this group (~27 %) is substantially higher than the MF-CHG (~20 %). Interestingly, the lipid levels and blood pressure levels of the LF-CHG and the MF-CHG are not well separated and their values lie within acceptable ranges.

For the second cohort of males, approximately 28 % (27/97) belongs to the LF-CHG (solid lines in Fig. 4, middle panels). Compared with the first cohort of males, the most salient feature of this cohort is the divergence of lipid measures and blood pressures. The LF-CHG has higher WC, BMI, PBF; higher LDL-C, triglyceride, and lower HDL-C; and higher SBP and DBP. Both the LF-CHG and MF-CHG show a decline in LDL-C, as compared to the relatively stable NHANES III trend (~95 mg/dl) , although the LF-CHG and MF-CHG are separated by a large gap of ~20 mg/dl.

For the third cohort of males, approximately 23 % (16/71) is identified as belonging to the LF-CHG (solid lines in Fig. 4, rightmost panels). Interestingly, the anthropometric and lipid measures continue to diverge and remain well apart for the LF-CHG and the MF-CHG while the blood pressure measures show convergence. The LF-CHG has substantially higher WC (~85 cm), BMI (~26 kg/m^2^), PBF (~22 %), lower HDL-C (~35 mg/dl), higher triglyceride levels (~140–160 mg/dl), and higher LDL-C (~100 mg/dl) than the MF-CHG. The elevated risk in this group seems to lie in its high level of triglyceride (>130 mg/dl, or high-level) as well as its consistent low level of HDL-C at <40 mg/dl.

## Discussion

Since the beginning of Project HeartBeat! in 1991, obesity in the United States, especially in children and adolescents, remains a prominent health concern [37, 38]. Early prevention may be strengthened when the relationship between obesity and cardiovascular risk factors can be clearly delineated, as these factors develop in childhood and adolescence [16, 23]. In this study, principal component functional curve analysis methods were used to summarize key features of trajectories of multiple longitudinal measurements. The principal components were then used to identify high and low risk groups (respectively labeled LF-CHG and MF-CHG) from both the male and female cohorts. The high risk groups had greater waist circumference, body mass index, and percent body fat as well as elevated LDL cholesterol and triglyceride levels, and lower HDL cholesterol levels. The risk profiles also revealed patterns of convergence and divergence across the high and low risk groups as a function of age.

Some general trends can also be observed by examining the functional curves across the cohorts. These observations have clinical and public health practice implications. For example, there are substantial age and gender differences in the trajectories across the risk groups MF-CGH and LF-CHG. Some measures such as DBP for females were not discriminating and they may not be useful to determine trajectories or characterize cardiovascular risk groups. It is interesting to note that for females, all lipid levels tend to show convergence between the LF-CHG and MF-CHG across the three age cohorts, whereas those for males all tend to show strong divergence. Our multi-variable analysis shows differences in HDL-C as well as LDL-C for the two risk groups begin at <3 mg/dl in cohort one and ends at >15 mg/dl in cohort three. Differences in the males’ triglyceride levels begin at <10 mg/dl at age 8 and end at >60 mg/dl at age 17. The dynamic in these indicators may provide clues to identifying important developmental periods for intervention.

The findings from the current study can also be used to inform future studies. For example, the identification of the LF-CHG could be used to define an exposure group in longitudinal studies for following up manifestations of heart disease or subclinical measures such as carotid intima-media thickness. Previous research identified important measures that were predictive of cardiovascular problems later on in life. The Muscatine Study [7, 9] suggested lipid and blood pressure levels at younger age were strong predictors of values later in life. The Princeton Lipid Research Clinics Follow-up Study found that 4.0 % of 771 children 6–19 years of age and followed for 25 years had the metabolic syndrome. Sixty-eight percent of those with the pediatric metabolic syndrome had the metabolic syndrome as adults and 19.4 % with the pediatric metabolic syndrome had CVD as adults, compared to 1.5 % for subjects without the pediatric metabolic syndrome [39]. Findings from this study point to early manifestation of cardiovascular risk in younger age and provide quantitative information about prevalence and trends. These findings could result in earlier interventions for reducing or delaying adverse metabolic changes and improving cardiovascular outcomes.

The study has limitations. First the study is limited by the lack of cohort data that span across all three age groups. The analysis was limited to stratification by gender and age-group. We have not further evaluated race because of sample size concern. Additionally, unlike NHANES III, the Project HeartBeat! sample is not a representative national sample. Finally, the study sample size is relatively small, especially for the LF-CHG. Because of this concern, results from this study may not be sufficiently robust to generalize to larger populations. The study is descriptive and the conclusion should not be viewed as definitive.

## Conclusions

Despite the aforementioned limitations, this study has strengths in its concurrent examination of a profile of cardiovascular risk factors including anthropometric, lipid, and blood pressure measures in several cohorts of boys and girls in their developmental stages. The results are unique in its simultaneous depiction of trajectories of the risk factors over time for a high-risk and a low-risk group, as well as the national norm, all by age group and gender. The findings could be used as important reference for future studies of cardiovascular health in adolescents. Our findings also suggest that male and female show rather distinct patterns in the trajectories, and lipid measure such as HDL-C and LDL-C show strong patterns of divergence in the risk groups while other variables such as blood pressures appear to be less discriminating.

## Abbreviations

AIC: akaike information criterion
BMI: body mass index
CVD: cardiovascular disease
DBP: diastolic blood pressure
FPC: functional principal component
HDL-C: high density lipoprotein cholesterol
LDL-C: low density lipoprotein cholesterol
LF-CHG: less favorable cardiovascular health group
MEC: mobile examination center
MF-CHG: more favorable cardiovascular health group
NHANES: National Health and Nutrition Examination Survey
PBF: percent body fat
SBP: systolic blood pressure
T2DM: type 2 diabetes mellitus
TG: triglycerides
WC: waist circumference

## References

[1] Reinehr T, Wunsch R, Putter C, Scherag A (2013). Relationship between carotid intima-media thickness and metabolic syndrome in adolescents. J Pediatr.

[2] Chaput JP, Lebalnc C, Perusse L, Despres JP, Bouchard C, Tremblay A (2009). Risk factors for adult overweight and obesity in the Quebec Family Study: have we been barking up the wrong tree?. Obes.

[3] Vanhala M, Vanhala P, Kumpusalo E, Halonen P, Takala J (1998). Relation between obesity from childhood to adulthood and the metabolic syndrome: population based study. BMJ.

[4] Vanhala MJ, Vanhala PT, Keinänen-Kiukaanniemi SM, Kumpusalo EA, Takala JK (1999). Relative weight gain and obesity as a child predict metabolic syndrome as an adult. Int J Obes Relat Metab Disord.

[5] Berenson GS (1986). Ed. Causation of cardiovascular risk factors in children. Perspectives on cardiovascular risk in early life.

[6] Berenson S (1978). Childhood risk factors predict adult risk associated with subclinical cardiovascular disease: he Bogalusa Heart Study. Circulation.

[7] Mahoney LT, Lauer RM, Lee J, Clarke WR (1991). Factors affecting tracking of coronary heat diseae risk factors in children. The Muscatine Study. In: Williams CL, Wynder EI., editors. Hyperlipidemia in childhood and the development of atherosclerosis. Ann NY Acad Sci.

[8] McGill HC, MacMahan CA (1998). Pathological Determinants of Atherosclerosis in Youth (PDAY) Research Group. Determinants of atherosclerosis in the young. Am J Cardiol.

[9] Thomsen M, Nordestgaard BG (2004). Myocardial infarction and ischemic heart disease in overweight and obesity with and without metabolic syndrome. JAMA Intern Med.

[10] Kuller LH, Lecher FG, Frundy SM, Hayman L (1999). The epidemiology of obesity in adults in relationship to cardiovascular disease. Obesity: impact on cardiovascular disease.

[11] Harrist RB, Dai S (2009). Analytic methods in project HeartBeat!. Am J Prev Med.

[12] Dai S, Fulton JE, Harrist RB, Grunbaum JA, Steffen LM, Labarthe DR (2009). Blood lipids in children: age-related patterns and association with body-fat indices: Project HeartBeat!. Am J Prev Med.

[13] Fulton JE, Dai S, Grunbaum JA, Boerwinkle E, Labarthe DR (2009). Effects of apolipoprotein E genotype on blood cholesterol in adolescent females. Am J Prev Med.

[14] Dai S, Harrist RB, Rosenthal GL, Labarthe DR (2009). Effects of body size and body fatness on left ventricular mass in children and adolescents: Project HeartBeat!. Am J Prev Med.

[15] Eissa MA, Wen E, Mihalopoulos NL, Grunbaum JA, Labarthe DR (2009). Evaluation of aap guidelines for cholesterol screening in youth: project HeartBeat!. Am J Prev Med.

[16] Labarthe DR, Dai S, Day RS, Fulton JE, Grunbaum JA (2009). Findings from Project HeartBeat!: their importance for CVD prevention. Am J Prev Med.

[17] Day RS, Fulton JE, Dai S, Mihalopoulos NL, Barradas DT (2009). Nutrient intake, physical activity, and CVD risk factors in children: project HeartBeat!. Am J Prev Med.

[18] Steffen LM, Dai S, Fulton JE, Labarthe DR (2009). Overweight in children and adolescents associated with TV viewing and parental weight: project HeartBeat!. Am J Prev Med.

[19] Fulton JE, Dai S, Steffen LM, Grunbaum JA, Shah SM, Labarthe DR (2009). Physical activity, energy intake, sedentary behavior, and adiposity in youth. Am J Prev Med.

[20] Altwaijri YA, Day RS, Harrist RB, Dwyer JT, Ausman LM, Labarthe DR (2009). Sexual maturation affects diet–blood total cholesterol association in children: project HeartBeat!. Am J Prev Med.

[21] Labarthe DR, Dai S, Fulton JE, Harrist RB, Shah SM, Eissa MA (2009). Systolic and fourth- and fifth-phase diastolic blood pressure from ages 8 to 18 years: project HeartBeat!. Am J Prev Med.

[22] Eissa MA, Dai S, Mihalopoulos NL, Day RS, Harrist RB, Labarthe DR (2009). Trajectories of fat mass index, fat free–mass index, and waist circumference in children: project HeartBeat!. Am J Prev Med.

[23] Labarthe DR, Dai S, Harrist RB (2009). Blood lipids, blood pressure, and BMI in childhood and adolescence: background to project HeartBeat!. Am J Prev Med.

[24] Labarthe DR, Dai S, Day RS, Day RS (2009). Project HeartBeat!: concept, development, and design. Am J Prev Med.

[25] Ramsay JO, Silverman BW (2005). Functional data analysis.

[26] Jones MC, Rice JA (1992). Displaying the important features of large collections of similar curves. Am Statist.

[27] Yao F, Müller HG, Wang JL (2005). Functional linear regression analysis for longitudinal data. Ann Statist.

[28] Hagenaars JA, McCutcheon AL (2002). Applied latent class analysis.

[29] US Department of Health and Human Services, National center for health statistics (1996). The Third National Health and Nutrition Examination Survey (NHANES III, 1988 – 1994).

[30] Chumlea WC, Guo SS, Kuczmarski RJ (2002). Body composition estimates from NHANES III bioelectrical impedance data. Int J Obes Relat Metab Disord.

[31] Ogden CL, Li Y, Freedman DS (2011). Smoothed percentage body fat percentiles for US children and adolescents, 1999–2004.

[32] Kuczmarski RJ, Ogden CL, Guo SS (2002). 2000 CDC growth charts for the United States: Methods and development. National Center for Health Statistics. Vital Health Stat.

[33] McDowell MA, Fryar CD, Ogden CL (2009). Anthropometric reference data for children and adults: United States, 1988–1994. National Center for Health Statistics. Vital Health Stat.

[34] National High Blood Pressure Education Program Working Group on High Blood Pressure in Children and Adolescents (2004). The fourth report on the diagnosis, evaluation, and treatment of high blood pressure in children and adolescents. Pediatr.

[35] Expert Panel on Integrated Guidelines for Cardiovascular Health and Risk Reduction in Children and Adolescents (2011). Expert panel on integrated guidelines for cardiovascular health and risk reduction in children and adolescents: summary reports. Pediatr.

[36] Cleveland WS (1979). Robust locally weighted regression and smoothing scatterplots. J Am Statist Assoc.

[37] Ogden CL, Carroll MD, Curtin LR, McDowell MA, Tabak CJ, Flegal KM (2006). Prevalence of overweight and obesity in the United States, 1999–2004. JAMA.

[38] Ogden CL, Carroll MD, Kit BK, Flegal KM (2014). Prevalence of childhood and adult obesity in the United States, 2011–2012. JAMA.

[39] Morrison JA, Friedman LA, Gray-McGuire C (2007). Metabolic syndrome in childhood predicts adult cardiovascular disease 25 years later: the Princeton lipid research clinics follow-up study. Pediatrics.

